# A network of empirical ethics teams embedded in research programmes across multiple sites: opportunities and challenges in contributing to COVID-19 research and responses

**DOI:** 10.12688/wellcomeopenres.17548.1

**Published:** 2022-02-10

**Authors:** Nothando Ngwenya, Jennifer Ilo Van Nuil, Deborah Nyirenda, Mary Chambers, Phaik Yeong Cheah, Janet Seeley, Primus Chi, Lindiwe Mafuleka, Busisiwe Nkosi, Dorcas Kamuya, Alun Davies, Mira L Schneiders, Noni Mumba, Siphephelo Dlamini, Nicola Desmond, Vicki Marsh, Dinnah Rippon, Michael Parker, Sassy Molyneux

**Affiliations:** 1 Africa Health Research Institute, Durban, South Africa; 2 University of KwaZulu Natal, Durban, South Africa; 3 Oxford University Clinical Research Unit, Ho Chi Minh, Vietnam; 4 Nuffield Department of Medicine, University of Oxford, Oxford, UK; 5 Malawi-Liverpool-Wellcome Clinical Research Programme, Blantyre, Malawi; 6 Mahidol Oxford Tropical Medicine Research Unit, Bangkok, Thailand; 7 London School of Hygiene & Tropical Medicine, London, UK; 8 KEMRI-Wellcome Trust Research Programme, Kilifi, Kenya; 9 Ethox, University of Oxford, Oxford, UK; 10 Oxford Pandemic Sciences Institute, University of Oxford, Oxford, UK

**Keywords:** COVID-19, embedded research, Low and Middle Income Countries, health research, ethics, network

## Abstract

Covid-19 continues to teach the global community important lessons about preparedness for research and effective action to respond to emerging health threats.  We share the COVID-19 experiences of a pre-existing cross-site ethics network-the Global Health Bioethics Network-which brings together researchers and practitioners from Africa, Europe, and South east Asia. We describe the network and its members and activities, and the work-related opportunities and challenges we faced over a one-year period during the pandemic. We highlight the value of having strong and long-term empirical ethics networks embedded across diverse research institutions to be able to: 1) identify and share relevant ethics challenges and research questions and ways of ’doing research’; 2) work with key stakeholders to identify appropriate ways to contribute to the emerging health issue response – e.g. through ethics oversight, community engagement, and advisory roles at different levels; and 3) learn from each other and from diverse contexts to advocate for positive change at multiple levels. It is our view that being both embedded and long term offers particular opportunities in terms of deep institutional and contextual knowledge and relationships with and access to a wide range of stakeholders in place. Being networked offers opportunities to draw upon a wide range of expertise and perspectives operating at multiple levels, and to bring together internal and external perspectives (i.e. different positionalities). Long term funding means that the people and resources are in place and ready to respond in a timely way. However, many tensions and challenges remain, including difficulties in negotiating power and politics regarding roles that researchers and research institutions play in an emergency, and the position of empirical ethics activities in programmes of research more specifically. We discuss some of these tensions and challenges, and consider the implications for our own and similar networks in future.

## Disclaimer

The views expressed in this article are those of the authors. Publication in Wellcome Open Research does not imply endorsement by Wellcome.

## Key messages

Empirical ethics, which combines conducting empirical—often qualitative—(social) research with philosophical analysis and reflection, can contribute to identification, consideration and addressing of the moral dimensions and practical ethics of pandemic responses, including research.

We share our COVID-19 experiences as a pre-existing cross-site empirical ethics network, called the Global Health Bioethics Network, which brings together researchers and practitioners from Africa, Europe, and South east Asia.

We highlight the value of having strong and long-term empirical ethics networks embedded across diverse research institutions in order to share ideas about emerging issues, and develop appropriate responses, including through conducting empirical research and playing advisory roles at institutional, regional and global levels. Such networks have the potential to contribute to negotiating persisting tensions and challenges, including regarding power and politics in institutional responses, and the position of empirical ethics in multi-disciplinary research programmes; and can make important contributions to pandemic preparedness.

## Background

Large scale epidemics demand a wide range of responses, from preparedness activities, through identification of ‘entry points’ for emergency interventions and coordination of multiple control activities and actors, to dealing with the aftermath ^1^. Throughout these responses, national and local health systems should ideally be supported not only to be able to absorb the shock of the epidemic, but also to introduce system changes that contribute to positive transformation, such that those systems are better prepared for future similar epidemics ^2^. Research, including social science research, can play a crucial role in feeding into and evaluating epidemic responses. Empirical ethics, which combines doing empirical—often qualitative—(social) research with philosophical (normative ethical) analysis and reflection, can contribute to identification, consideration and addressing of the moral and practical ethical dimensions of the research and response ^3^. Engagement with these issues is essential to the success and sustainability of epidemic response and preparedness ^4^.

During the Ebola and Zika pandemics, there were concerns that the social sciences, including empirical ethics, were neglected relative to epidemiology and basic science ^5– 8^. Limited funding meant that much of the research was too reactive and rapidly conducted to contribute meaningfully to the response, in turn perpetuating low prioritization of social science research. The empirical ethics research that was conducted highlighted the practical and ethical challenges of producing quality data during a pandemic, including logistical difficulties and concerns of power dynamics between local and international actors, and between researchers and research participants and communities. These past epidemics also shone a light on the need to strengthen governance and accountability of the public health response and research during epidemics, including ensuring meaningful involvement of relevant stakeholders, not least affected communities ^1, 4, 8, 9^.

Given the scale and nature of the COVID-19 pandemic, the focus of global and national response has been on clinical medicine and public health activities. From the outset - in part learning from past epidemics - research and implementation networks advocated for local contexts to be considered in public health responses ^10, 11^. It was emphasized that failure to see outbreaks as political and socioeconomic emergencies, as well as public health emergencies, risked the pandemic response doing more harm than good (see for example materials on the Behavioural, Environmental, Social and Systems Interventions ( BESSI) and Training And Resource Centre ( TARSC) sites). Social justice concerns specifically were highlighted, with COVID-19 being seen to be deepening socio-economic inequities locally, nationally and globally ^12, 13^. The urgency of conducting research was recognized as a moral issue, in line with the WHO’s position that “ *During an infectious disease outbreak there is a moral obligation to learn as much as possible as quickly as possible, in order to inform the ongoing public health response, and to allow for proper scientific evaluation of new interventions being tested*” ^14^. Significant COVID-19 specific research funding was announced, including specifically for social science research, which could also allow for empirical ethics and engagement with communities (see for example SSRC, Wellcome Trust and African Academy of Sciences). Nevertheless, the pandemic brought new challenges for research and engagement, including far more widespread travel and interaction restrictions than experienced in previous epidemics. Also, some have argued that ethicists and policymakers focused too little on the ethics of research; that is, the normative work required around risk/benefit analyses, issues concerning informed consent, and privacy considerations as well as empirical analyses of data on patient and public priorities, and the real-world barriers faced by researchers and oversight committees ^14^.

In this paper we share our experiences during COVID-19 as a pre-existing cross-site empirical ethics network, called the Global Health Bioethics Network (GHBN). We describe the ways in which GHBN members were already embedded across diverse research institutions in Africa, Europe, and South east Asia, and the opportunities and challenges we faced in contributing to COVID-19 responses and conducing COVID-19 empirical ethics research.

## Network description and methodological approach

### What is the Global Health Bioethics Network

The Global Health Bioethics Network (GHBN) is a collaborative partnership bringing together interdisciplinary ethics, engagement and social science teams based at the five Wellcome Africa and Asia Research Programmes (AAPs) in Kenya, Malawi, South Africa, Thailand, Vietnam, and the Ethox Centre in the UK. GHBN was established in 2012 through a strategic award provided by the Wellcome Trust. The AAPs themselves were established in the 1970’s, 80’s and 90’s with a focus on medical research into tropical diseases relevant to their regions (such as HIV, TB, malaria, and others). All have evolved into large inter-disciplinary research programmes over the decades, with numerous strong regional and global collaborations, and all have strong links with, or are embedded within, their respective national health and research systems. For example, the Oxford University Clinical Research Unit (OUCRU) in Viet Nam was co-founded in 1991 by the Hospital for Tropical Diseases (which is under the direction of the Ministry of Health) and is located in the hospital grounds in a building jointly funded by the Vietnamese government and Wellcome Trust. The Malawi-Liverpool-Wellcome Trust Clinical Research Programme (MLW), set up in 1995, is an integral part of the University of Malawi College of Medicine, and has a specific Policy Unit that partners with key stakeholders in the health sector in Malawi and internationally. All the AAPs have staff involved in advisory committees locally, nationally, regionally and globally. All also have a long history of developing and implementing community engagement activities linked to their research programmes.

The GHBN has its origins in the AAPs’ increasing interest in and commitment to addressing the ethical aspects of their research programmes and of ethical questions in relation to global health more broadly. Embedded in the Wellcome Africa and Asia Programmes, and in collaboration with the Ethox Centre at the University of Oxford, the GHBN has three main aims: (i) to promote and support the identification of ethical issues and ethical reflection across the AAPs and their research partners in a wide range of other low-resource settings; (ii) to build the capacity of the AAPs and their partners to identify and address ethical issues in their research, through post-docs, PhDs, bursaries, and other training activities; and, (iii) to facilitate and support ethics research on pressing ethical issues arising in the scientific research programmes of the AAPs and on key questions in global health.

The members of the GHBN come from many different backgrounds. While some are trained in philosophy and research ethics, others are sociologists, psychologists, human geographers and anthropologists as well as clinicians, and public health and community engagement specialists. Most members based in the AAPs have primary roles either as research staff or community engagement personnel, with many wearing several of these professional hats. All members interact in their daily lives with a wide range of research, health system and community stakeholders, and all are actively engaged in research and engagement activities related to global health ethics. As part of their academic citizenship, members are involved in a wide range of activities including teaching, mentorship, journal editing and membership of research ethics and oversight committees as well as review boards and panels for research and international development funders.

The ways in which GHBN members are embedded within their own institutions differs, in part related to the varying ways in which social science, ethics support, and community engagement activities have evolved within those institutions; an evolution influenced at least in part by GHBN. For example, at the KEMRI-Wellcome Trust Research Programme (KWTRP), coordination of engagement activities from 2005 was initially led by a small social science group, and run as an action research activity, until the increased scale and breadth of both the engagement and social science research led to each ‘area’ functioning relatively independently. Most KWTRP GHBN members are now part of a Health Systems Research Ethics Department, with researchers conducting empirical ethics, biosocial and health policy and systems research, often in collaboration with clinical, epidemiological and bioscience colleagues in other departments. A somewhat different evolution is seen in the Thailand AAP, also known as the Mahidol Oxford Tropical Medicine Research Unit (MORU). Community engagement activities had always been conducted for studies in MORU, but it was not until the GHBN was established that this work begun to be published, contributing to the formalization of a programme of bioethics and engagement research, and ultimately the establishment of a new group called “Bioethics & Engagement” in 2015. This group has embedded ethics and engagement work into many projects at MORU such as the mass antimalarial drug administration project in the Greater Mekong Subregion ^15, 16^, in many cases with ring-fenced budgets.

### Covid-19 and how it unfolded in GBHN settings

**Figure 1.**
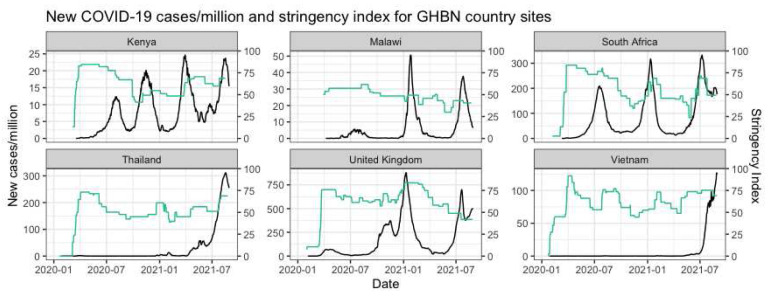
New COVID-19 cases/million and stringency index for GHBN country sites. Green line represents the stringency index and the black line represents the COVID-19 cases/million (add cite) Sources: Hale *et al.*, 2021 and Ritchie *et al.*, 2020. • *Hale, Thomas, Noam Angrist, Rafael Goldszmidt, Beatriz Kira, Anna Petherick, Toby Phillips, Samuel Webster, Emily Cameron-Blake, Laura Hallas, Saptarshi Majumdar, and Helen Tatlow. 2021. “A Global Panel Database of Pandemic Policies (Oxford COVID-19 Government Response Tracker).” Nature Human Behaviour 5(4):529–38.* • *Ritchie, Hannah, Edouard Mathieu, Lucas Rodes-Guirao, Cameron Appel, Charlie Giattino, Esteban Ortiz-Ospina, Joe Hassell, Bobbie Macdonald, Diana Beltekian, and Max Roser. 2020. “Coronavirus Pandemic (COVID-19).” OurWorldInData.Org.*

By the time COVID-19 began to be recognized across the world as a global health emergency, the GHBN had already been in place for nine years. The first COVID-19 cases were reported in different months across the main countries involved, with Thailand being the first country to record a case, followed by Vietnam, UK, South Africa, Kenya and Malawi ( Figure 1). Notable was the very different initial responses taken by national governments in our respective countries. In UK for example, there was initial hesitation to introduce lockdown measures, and re-assurance of the public that COVID-19 was relatively mild and ‘flu like’, until mortality rates soared in April/May 2020 ^17^. In contrast, in South Africa and in Kenya, as in many African countries, there were swift responses in March 2020 into hard lockdowns with the first cases identified in the two countries; responses that were initially praised in the local and regional media as highly effective. Inevitably, as the pandemic has progressed over time and space, there have been shifts in the epidemiology and responses, with each country having experienced one to four distinct waves.

As with so many people around the world, GHBN network members had to start to work from home with little notice when lockdowns were introduced in their countries. By April 2020, almost all countries were in varying degrees of lockdown, and the majority of network members were working from home. Almost all non-COVID-19 studies across all sites were required by national governments and ethics review committees, or by institutional leads, to close down, ‘hibernate’, or reduce to minimal carefully socially distanced activities, with exceptions being studies conducted online. As lockdowns hardened, only studies where participants or family members would be put at risk by a discontinuation or break in the research activities were permitted with even minimal interaction (such as clinical trials where safety monitoring was still essential). The latter monitoring moved online wherever possible.

### Tracking opportunities and challenges

Early on in the pandemic we recognized the huge ethical implications of COVID-19 for health systems, communities and for health research in our different contexts, and the potential value and responsibility of having a network such as ours with diverse members already embedded in very different institutions in different settings. However, we also began to face a wide range of concerns and frustrations linked to our embedded positions. We recognized the pressing importance of exploring the ethical issues associated with COVID-19 related research, medical interventions, social distancing and other non-pharmaceutical measures such as contact tracing. As recognition of shared issues, worries and the need to learn from one another became clear across the network, as well as our interest and responsibility to learn from and contribute to responses locally, nationally and globally, we began to organize regular online monthly meetings. These meetings typically lasted one to two hours, were relatively informal and relaxed, and usually involved each site outlining ongoing activities and issues in order to prompt discussion and learn from each other’s experience.

In the following sections we describe the opportunities and challenges we have faced and discussed over the year and a half since establishing the meetings, related to four inter-related areas including the immediate and direct responsibilities with regards to supporting frontline colleagues; advisory roles in COVID-19 responses; engaging meaningfully with community representatives and broader publics; and conducting both empirical ethics and ethical analysis to contribute to ensuring ethical considerations are embedded in the pandemic response and to highlight other impacts and issues. These opportunities and challenges were identified through reviewing the minutes and notes from our regular meetings over a period of one year (April 2019 to July 2020), and two cross-network discussions each of two hours (July 2020 and July 2021) where we shared the content of this paper and sought further reflection.

## Opportunities and challenges faced by GHBN members

### Immediate and direct responsibilities with regards to supporting frontline colleagues

Emerging early on in the pandemic was a concern about the health and well-being of our research team members (including fellow researchers of varying levels of seniority, field staff, community liaison staff, students, and early career researchers) and of health workers, community representatives, colleagues and friends.

For staff employed by our own institutions, we were fortunate that most institutions were able to continue to pay salaries while staff worked at home. However, we all recognized the very different home environments that we were all working in. Although there were efforts to ensure that all staff who needed it had access to computers and modems that could be used at home, and pre-paid data packages paid for, we were never able to resolve the issues that many – but by no means all – staff were facing. For example, many GHBN members and their colleagues or people they were responsible for were facing challenges with electricity and internet access, inadequate or overcrowded accommodation, and multiple disruptions and demands from family members. Other colleagues were relatively isolated, and with some it was difficult to judge well-being through phone and online engagements only. Regular communication systems and structures were set up, and new activities organized online such as journal clubs and regular informal teas or coffees in an effort to maintain a sense of community. Such initiatives have shifted over time, and as the pandemic has extended. However, and as noted globally, such approaches have worked better for some staff than others, and it remains difficult to ensure that those who need support are receiving it.

GHBN members wished to support colleagues in health systems and communities, but often felt powerless to do so in a formal and systematic way. Many of us also felt a weight of responsibility linked to a recognition that we were in a relatively privileged position, albeit variously so, in terms of physical, employment security, as well as in relation to access to healthcare and, ultimately, vaccination. There were some opportunities to draw on our past activities and relationships, as well as our recognition of the issues being faced, to share the issues we were learning about across our institutions and with wider audiences. Thus, for example in Kenya we were able to draw on past long-term health systems work ^18– 21^, and on-going interactions ^2, 22^, to develop a set of policy briefs, including on the COVID-19 implications for health system resilience, and on the need for better practical and emotional support for frontline health workers. The latter was discussed in a wider webinar, also involving GHBN colleagues in Vietnam. Another example is from Malawi where we contributed to a policy brief on strategies for enhancing community engagement in Covid-19 case-finding, contract tracing and case referral. Nevertheless, these initiatives inevitably felt piecemeal and inadequate.

### Playing an advisory role in COVID-19 responses locally and globally

Many GHBN members felt an immediate responsibility and desire to contribute to public debate, and to local, national, regional and global responses. Community engagement and research related opportunities and challenges are described next, but, more broadly, many members began to play a range of different advisory and review roles.

As noted above, GHBN members come from diverse disciplinary backgrounds, and play multiple roles and wear many professional hats within their own institutions and communities, nationally and globally. Many were therefore either already in a position, or were soon offered opportunities, to take on diverse formal advisory/review roles. Illustrations of types of roles played include GHBN members sitting on advisory groups for the South African and UK governments (through a national communications cluster and the Scientific Advisory Group for Emergencies (SAGE), respectively), and, at regional level, the WHO-Afro African Advisory Committee on Health Research and Development (AACHRD). At the global level, GHBN members sit on the WHO Task force on social science research, the WHO Task Force on Good Participatory Practices in Emerging Pathogens (GPP-EP), the WHO Ethics Advisory Group on COVID-19, PREPHEN (a WHO led preparedness, research and response network to support epidemic ethics) and various working groups as part of the COVID-19 Clinical Research Coalition such as the Data Sharing Working Group ^23^, and the Ethics Working Group.

WHO task forces and networks have contributed to identifying COVID-19 related research priorities globally, and the development of key documents to guide Good Participatory Practice to facilitate Covid-19 clinical trials ^24^. In all cases, task force members specifically drew on GHBN inputs in terms of ideas and documents, including the policy briefs mentioned in the previous section. Preceding COVID-19, members of the GHBN also sat on the Nuffield Council on Bioethics Working Group on Research in Global Health Emergencies: ethical issues, the report of which was published in January 2020 ^4^.

Throughout the COVID-19 pandemic, these roles have been important opportunities to share learning, ideas and outputs from our own sites, and from the discussions and ideas both across the GHBN and also with the wider world. In so doing we sought to have a positive impact on the unfolding context, although associated dilemmas included: 1) our power as individuals within these groups and committees, and the power of those fora to make a meaningful and lasting impact; 2) our recognition of the unprecedented and constantly shifting nature of the pandemic across time and space and therefore our own concerns about the relevance of our past data and knowledge; 3) some of the opportunities and challenges associated with community and public engagement, and conducting empirical research, described next.

### Responsibilities and approaches to engaging with community members

A central component of much of the empirical ethics work across our institutions, which over time has been valuably supported, critically reviewed, and deepened through the GHBN, is engagement with community members and broader publics ^25– 35^. An immediate potential opportunity and need under COVID-19 was to work with established community engagement networks with whom we had long term relationships in order to: 1) learn about community members’ and the broader publics’ priorities and concerns in relation to the response, and to communicate these up through institutions and to local, national and global policy makers and influencers through our wider networks in order to inform locally tailored information giving and action; 2) seek community members’ advice and inputs on how to manage closure or appropriate continuation of non-COVID-19 related studies; and 3) engage community members on prioritizing and planning COVID-19 studies.

Across our different institutions we were able to work towards these different aims in different ways. In terms of feeding community member and broader public priorities and concerns into information giving and action, at the most basic level GHBN members’ institutions in all countries were able to draw upon their networks and use their resources to support the national health teams to distribute government developed IEC materials. In some sites (for example KWTRP), all other information sharing and public engagement by institutions and organisations was initially stopped, in order to minimise public confusion and misinformation. National requests for support from researchers focused instead on COVID-19 testing and advisory roles based on epidemiological, health systems and existing socio-behavioural data and literature. In most of the other sites, research groups were able to be more directly involved in local information sharing about COVID-19. In MLW and AHRI for example, engagement staff assisted the District Health Office and National Defence Force in organising mass awareness campaigns and meetings, including through implementing education sessions at markets, and working with traditional health practitioners and tribal/local leaders who have an influence in communities. They were also able to work with ministry of health colleagues to use social media, theatre shows, mobile vans, radio and TV stations to reach large numbers of people to provide information and address rumours and concerns. In OUCRU, GHBN members conducted media monitoring in Vietnam, Indonesia and Nepal. They started monitoring local social media and news platforms early in the pandemic (April 2020), and by continuing to do so through vaccine initiation and roll-out (starting January 2021), were able to track and respond to emerging COVID-19 anxieties with locally tailored, evidence-based social media posts. At MORU, GBHN members based on the Thai-Myanmar border engaged with Karen and Burmese migrant workers who faced language barriers regarding COVID-19 related information conveyed in Thai by the Thai authorities.

With regards to seeking community members’ advice and inputs on how to manage the often sudden closure or continuation of non-COVID-19 related studies, community engagement activities themselves often either had to stop, or to shift online, in some cases with very little notice. In Kenya for example there was little opportunity before face-to-face meetings with community representatives were halted to develop and test new ways of engaging. Over time, online approaches were developed across sites such as setting up WhatsApp groups and phone-based interactions and discussions. Although this allowed some continuation of community engagement activities, including to develop and plan COVID-19 specific studies, many community members have poor access to appropriate phones, if they have any phones at all, and are living in areas with intermittent electricity and poor network coverage. Even when data packages are provided for community representatives, and there is electricity, technological challenges remain in terms of connectivity, influencing which community representatives can connect online with community engagement staff. . Thus there were concerns that existing challenges in hearing the voices of the most vulnerable and least connected community members would be exacerbated.

Where online interactions and face-to-face meetings were possible with community representatives, engagement staff faced dilemmas in how to respond to the numerous issues raised with regards to their own and other community members’ access to testing and health care, as well as reports about socio-economic stresses and strains and health consequences resulting from COVID-19 fears and lockdowns. Although community engagement staff were able to respond with government approved information, many reported feeling relatively helpless to respond, and in fact often faced similar anxieties and concerns themselves (as described further below). GHBN members drew on their conversations with community members, as well as on their reflections with each other and with others to write commentaries and blog pieces to raise awareness of the issues being faced, and to feed into advisory groups and webinars. Through these mechanisms, GHBN members highlighted the importance of community engagement, as well as some of the opportunities, challenges, and social justice concerns with national and global responses (see for example ^2, 36– 38^).

### Need and ability to conduct empirical ethics studies

Many of us were keen to move beyond the above engagement activities to build on our existing relationships, networks and platforms to initiate COVID-19 studies, or to add a COVID-19 lens into existing studies. We were interested both in stand-alone studies (for example social justice implications of the response, how to give voice to the needs of the most vulnerable groups, or moral/ethical dilemmas being experienced by research and frontline staff and highlighting support needs), as well as empirical ethics elements built into inter-disciplinary clinical and epidemiological studies that were rapidly being designed and implemented by colleagues (for example potential participants’ perceptions of COVID-19 and of COVID-19 studies). We recognised the potential of cross-site research, given our shared interests and the similarities and differences across sites in terms of the COVID-19 epidemic and responses, institutional histories, structures and foci, and socio-economic and political contexts. All our environments are also shaped – albeit in different ways - by intersecting structural influences of poverty, patriarchy, capitalism, racism, and colonialism, and most of our institutions have at least a historical dominance of biomedical approaches.

We had some unique opportunities with regards to developing, funding and conducting empirical ethics research. First, much of the community engagement we were involved in as described above was an opportunity to systematically document and track community priorities and concerns, as well as ethical dilemmas and concerns of frontline research and health system staff. Second, there was already a wide range of ongoing approved studies in place before COVID-19 was reported in countries, where a COVID-19 lens could be added, for example evaluating community engagement, exploring community treatment-seeking behavior, identifying frontline health worker priorities and concerns, examining system governance and oversight, and epidemiological and clinical studies. Adding a COVID-19 and community perspective lens to these studies was potentially feasible where interviews could be conducted online or following local and national social distancing rules. Online interviews, particularly for interviews seeking more than simple quantitative data, were most realistic to consider for longitudinal studies where relationships were already in place between researchers and participants. Third, we were able to design and conduct new studies, considering social distancing rules and requirements, and the potential for these to change over time, from the outset.

All amendments to studies and new studies clearly needed institutional and national ethics approval, following careful consideration of the full range of potential ethical issues (including risks/benefits, consent, confidentiality, and data safety/protection). In terms of funding, we were fortunate to have costed extensions offered automatically by one of the main funders in our institutions – Wellcome Trust - for salary time, including an extension of the GHBN. Thus, some studies could be conducted with relatively little additional funds. A valuable source of additional funds was a small, competitively run bursary scheme run by GHBN for early career research in LMICs. An example of an international study funded through a combination of mechanisms is provided in Box 1. As elsewhere, the social scientists involved in planning studies sometimes had to make methodological compromises to fit national or institutional rules (such as more on-line and less in-depth and deliberative work, leading to concerns about missing the voices of the most vulnerable), and sometimes worried about team members’ safety where face-to-face interviews were permitted. Our own dilemmas and discussions fed back into decision-making across our institutions and more widely, as described above.

**Box 1. Examples of funded international studies**

One example of a study that received both internal and external funding is the Social Science and Public Engagement Action Research in Vietnam, Indonesia and Nepal (SPEAR) study, initiated in June 2020. The primary aim of SPEAR was to explore the experiences and impact of COVID-19 and the public health response for healthcare workers and related staff from a variety of healthcare settings, as well as community members who were more impacted by COVID-19 and/or the response. The study was conducted in 12 sites across the three countries, each of which were facing different reported levels of COVID-19 and different responses. Each site had both healthcare worker and community participation and each country had representation from both rural and urban communities. For data collection, the team used media monitoring, questionnaires (self-administered online or paper and interview administered in person or on phone), in-depth interviews (in person and online), and digital diaries. In January 2021, the SPEAR study was expanded to include an additional focus on access to and perceptions of COVID-19 vaccination within all community sites involved in SPEAR. Another example of an externally funded proposal is a multi-site study – involving researchers based in Kenya, South Africa and Ghana - aimed at examining how research review and regulatory systems have responded to the high demand for COVID19 research, including how systems have been changed, the (ethical) issues raised, the opportunities and challenges from the perspective of different stakeholders, and if and how any challenges have been addressed.

A challenge faced by many GHBN members and colleagues for COVID-19 related work in many (but not all) sites was lengthy funding and especially ethics review processes. For example, although the SPEAR study ( Box 1) was approved within three months of submission, a different study in another country (an interview and observation-based study aimed at systematically exploring the ethics issues and dilemmas experienced under COVID-19), took 8 months from submission to final approval, primarily due to administrative delays and changes in regulatory processes. Further challenges for empirical ethics included, particularly early on in the pandemic, keeping pace with biomedical research, for which there was often a clearer and more immediate demand from national stakeholders, and ethics researchers not being involved in research planning meetings. Broader challenges included a relatively small group of people being aware of the unfolding requirements and issues within institutions, and a fast-evolving situation which was not easily shared across relevant colleagues. Findings from social science/empirical ethics studies that were prioritized and conducted have begun to be published and shared through our networks, we hope contributing to public and policy maker awareness about lived experiences and priorities and concerns on the ground ^13, 27, 39– 43^.

Finally, in addition to the empirically driven ethics research GHBN was able to undertake during the COVID-19 pandemic, some members of the network were also well-placed – because of their membership of GHBN, their embeddedness in COVID-19 related research initiatives, and their policy roles – to write a number of influential papers and blogs addressing key ethical questions relating to the impact of the pandemic and of responses to it.

## Discussion

The Global Health Bioethics Network is a strong and long-term social science and ethics network bringing together researchers and practitioners from Africa, Europe, and South East Asia. Being embedded and long-term has allowed us to interact in ways that are different to some of the concerns typically raised about ‘parachute’ style international collaborations ^5, 44^, where Northern researchers drop into Southern settings to undertake research without adequate attention to equitable treatment of Southern partners and without adequate understanding of local contexts. In response to the pandemic, this offered us particular opportunities, but as with many other researchers across the world, we also encountered many tensions and challenges.

As an existing network with established relationships, many of us discussed feeling able to share issues and concerns in a safe and supportive online environment. It was often referred to in discussions as a valuable form of ‘group therapy’ in a very difficult and uncertain time, like an informal debrief session (similar to but less formalized than as described in Molyneux *et al.* ^45^ and McMahon and Winch ^46^). All of us described feeling reassured through these discussions and the sense of community they created, and in some cases, there was validation by others that the issues and concerns we had were legitimate, even where our own institutions were unable to hear or engage with them.

The network also offered us intellectual and practical support to reflect upon and discuss our various responsibilities, not least balancing across our (sometimes competing) responsibilities to safeguard the wellbeing of staff and colleagues, engage with community representatives and other stakeholders, conduct timely and responsive research and offer relevant and appropriate advice at different levels (such as through the committees and advisory groups we sit on). Being embedded and long term offered us particular opportunities in terms of deep institutional and contextual knowledge and having existing relationships with and access to diverse stakeholders including within communities, health and research institutions and policy-makers. This gave us opportunities in terms of understanding and working with those with the power the make decisions in complex environments, and having established relationships in place as a platform for research and response support. Being networked offered us opportunities to draw upon a wide range of roles and expertise operating in multiple contexts, and to bring together perspectives internal and external to institutions (ie different positionalities ^47^). This in turn supported us to consider similarities and differences across contexts and gave us greater confidence to contribute to institutional, national and global discussion and debates (see for example resources shared on Global Health Bioethics and on The Global Health Network).

However, many tensions and challenges remain, as summarized in Table 1. In terms of conducting empirical research, we faced the challenge that has been shared elsewhere, of social sciences, including empirical ethics, sometimes being considered as ‘nice-to-have’ rather than as essential as clinical or epidemiological research, and therefore something that can wait until a less urgent or emergency situation to be planned, reviewed and conducted ^5– 8^. Where the value of social science is seen, we noted a preference for quantitative and representative data, and for rapidly produced research, as opposed to more in-depth and time-consuming qualitative data investigating context, values, beliefs, concerns, solutions, and lived experiences. The importance of conducting a range of research across timelines was highlighted from early on in the pandemic on social media and through research networks (see for example Marquette H, April 2020). However, this was challenging to implement in practice. We were all aware of and conflicted by - on the one hand - a desire to help with the response through research, and on the other a concern that the research that we were conducting should be relevant, appropriate and of high quality. A particular concern was being careful not to turn all of our attention to COVID-19, thereby undermining our ability to feed into and conduct essential research on other social and health issues in communities. Another concern expressed by some network members was not feeling experienced enough to contribute to arguments about how to think about ethical issues around COVID-19 related decision-making in ways that could usefully feed into institutional or national decision-making; an area we wish to continue to teach one another across the network.

**Table 1.** Tensions an issues faced.

Tension/issue	How tension was felt
*Social science vs ‘real’ science*	Social science is sometimes seen as non-essential research to do later, once the more critical epidemiological or clinical research is underway
*Quantitative vs qualitative* * social science*	Quantitative social science can be prioritized over more in-depth qualitative work, and studies with small sample sizes dismissed or not taken seriously (even when appropriate for the question, theoretically informed, and well implemented)
*Speed vs quality*	Producing findings ‘NOW’ vs learning in depth and in detail for future
*In-depth, inclusive learning*	‘What is ethical?’ and what is possible under physical distancing rules
*Community engagement vs* * social science*	Engagement and social science are always the same activities, even though there may be overlaps in people, methods, and interests
*Confidentiality vs impact*	Protecting participant/institution confidentiality vs maximizing the policy impact/uptake of research findings
*Immediate vs longer-term/* *structural issues*	Balancing dealing with immediate challenges/issues against tackling longer-term/structural issues such as having to seek funding primarily from high income countries
*Social science findings vs* * stakeholder interests*	Some findings can be uncomfortable for some research stakeholders ( *eg* research institution/ biomedical research team / government)… requires management of relationships

A related tension was similarities, differences and overlaps between community engagement and social science research, including empirical ethics. Engagement activities offer opportunities for, and can contribute to answering research questions, but the latter may have particular methodological and ethical considerations (for example regarding consent, benefits and confidentiality) as well as require different institutional and national approval processes to be in place. We had to consider these similarities, differences and their implications in all our activities on a case-by-case basis. For example, with the SPEAR study described above, the integration of engagement and social science provided the opportunity to use findings from engagement activities such as participant-led films or ‘digital diaries’ and media monitoring to inform the survey and interview tools at the early stages of the project ^13, 27^. During the social science data collection, especially related to vaccines, the engagement and social science teams met regularly so data from the interviews could inform engagement messages.

Across both engagement and research activities involving community members, there was a tension in ensuring that the voices of those who were potentially most vulnerable to the worst impacts of COVID-19 were heard, but that the activities themselves did not add to those vulnerabilities. Thus for example in KWTRP, the team/members considered building on relationships we already had with mothers of young children in rural and urban settings discharged from hospital (through 48, 49) to learn about their priorities and concerns regarding COVID-19. However, in GHBN and team meetings we agreed there was too much potential to inadvertently contribute to disadvantages and harms, both physical (COVID-19) and in terms of perceived responsibilities to act upon issues raised that could not be met. We had concerns about the potential harms for both those conducting the interviews (if households were visited) and for household members themselves (if interviews were conducted over the phone, or in a central place).

A final tension and challenge was a recognition that the institutions most centrally involved in the GHBN network are all relatively privileged compared to many others based in low-resource settings, and that GHBN members within those institutions were unequally involved in the regular COVID-19 related discussions (given differences across staff in terms of home living arrangements, access to electricity/power cuts, health and well-being and levels of work and home-related responsibility). Although the network activities and ways of working seek to challenge such inequities, our own interactions are also shaped – albeit in different ways - by intersecting structural influences of poverty, patriarchy, capitalism, racism, and colonialism that interplay to impact on us all differentially over time and space. As a network this is an area we are keen to continue to reflect upon and to contribute to ideas and activities aimed at positive transformation.

## Conclusion

A lesson from this difficult and unprecedented time is that embedded ethics has an important role to play in effective infectious diseases research and response. Notwithstanding the limitations, practical concerns, and difficulties outlined above, it is our strong view that an embedded ethics network that brings together ethicists and social scientists from a wide range of contexts in a sustainable, long-term, networked and globally distributed collaboration has the potential to be a crucial resource in pandemic preparedness, resilience, and response. The COVID-19 pandemic is not over and there is much valuable work for networks such as the GHBN to continue to do.

## Data availability

No data are associated with this article.
