# A network of empirical ethics teams embedded in research programmes across multiple sites: opportunities and challenges in contributing to COVID-19 research and responses

**DOI:** 10.12688/wellcomeopenres.17548.2

**Published:** 2023-01-27

**Authors:** Nothando Ngwenya, Jennifer Ilo Van Nuil, Deborah Nyirenda, Mary Chambers, Phaik Yeong Cheah, Janet Seeley, Primus Chi, Lindiwe Mafuleka, Busisiwe Nkosi, Dorcas Kamuya, Alun Davies, Mira L Schneiders, Noni Mumba, Siphephelo Dlamini, Nicola Desmond, Vicki Marsh, Dinnah Rippon, Michael Parker, Sassy Molyneux

**Affiliations:** 1 Africa Health Research Institute, Durban, South Africa; 2 University of KwaZulu Natal, Durban, South Africa; 3 Oxford University Clinical Research Unit, Ho Chi Minh, Vietnam; 4 Nuffield Department of Medicine, University of Oxford, Oxford, UK; 5 Malawi-Liverpool-Wellcome Clinical Research Programme, Blantyre, Malawi; 6 Mahidol Oxford Tropical Medicine Research Unit, Bangkok, Thailand; 7 London School of Hygiene & Tropical Medicine, London, UK; 8 KEMRI-Wellcome Trust Research Programme, Kilifi, Kenya; 9 Socio-Ecological Health Research Unit, Institute of Tropical Medicine, Antwerp, Belgium; 10 Ethox, University of Oxford, Oxford, UK; 11 Oxford Pandemic Sciences Institute, University of Oxford, Oxford, UK

**Keywords:** COVID-19, embedded research, Low and Middle Income Countries, health research, ethics, network

## Abstract

Covid-19 continues to teach the global community important lessons about preparedness for research and effective action to respond to emerging health threats. We share the COVID-19 experiences of a pre-existing cross-site ethics network-the Global Health Bioethics Network-which brings together researchers and practitioners from Africa, Europe, and Southeast Asia. We describe the network and its members and activities, and the work-related opportunities and challenges we faced over a one-year period during the pandemic. We highlight the value of having strong and long-term empirical ethics networks embedded across diverse research institutions to be able to: 1) identify and share relevant ethics challenges and research questions and ways of ’doing research’; 2) work with key stakeholders to identify appropriate ways to contribute to the emerging health issue response – e.g., through ethics oversight, community engagement, and advisory roles at different levels; and 3) learn from each other and from diverse contexts to advocate for positive change at multiple levels. It is our view that being embedded and long term offers opportunities in terms of deep institutional and contextual knowledge, existing relationships and access to a wide range of stakeholders. Being networked offers opportunities to draw upon a wide range of expertise and perspectives, and to bring together internal and external insights (i.e.drawing on different positionalities). Long term funding means that the people and resources are in place and ready to respond in a timely way. However, many tensions and challenges remain, including difficulties in negotiating power and politics in the roles that researchers and research institutions can and should play in an emergency, and the position of empirical ethics within research programmes. We discuss some of these tensions and challenges and consider the implications for our own and similar networks in future.

## Disclaimer

The views expressed in this article are those of the authors. Publication in Wellcome Open Research does not imply endorsement by Wellcome.

## Key messages

Empirical ethics, which combines conducting empirical—often qualitative—(social) research with philosophical analysis and reflection, can contribute to identification, consideration and addressing of the moral dimensions and practical ethics of pandemic responses, including the conduct of research.

We share our COVID-19 experiences as a pre-existing cross- site empirical ethics network, called the Global Health Bioethics Network, which brings together researchers and practitioners from Africa, Europe, and Southeast Asia.

We highlight the value of having strong and long-term empirical ethics networks embedded across diverse research institutions to share ideas about emerging issues and develop appropriate responses, including through conducting empirical research and playing advisory roles at institutional, regional and global levels. Such networks have the potential to contribute to negotiating persisting tensions and challenges, including regarding power and politics in institutional responses, and the position of empirical ethics in multi-disciplinary research programmes; In so doing, these networks can make important contributions to pandemic preparedness.

## Background

Large scale epidemics demand a wide range of responses, from preparedness activities, through identification of ‘entry points’ for emergency interventions and coordination of multiple control activities and actors, to dealing with the aftermath ^1^. Through- out these responses, national and local health systems should ideally be supported not only to be able to absorb the shock of the epidemic, but also to introduce system changes that contribute to positive transformation, such that those systems are better prepared for future similar epidemics ^2^. Research, including social science research, can play a crucial role in feeding into and evaluating epidemic responses. Empirical ethics, which combines doing empirical—often qualitative—(social) research with philosophical (normative ethical) analysis and reflection, can contribute to identification, consideration and addressing of the moral and practical ethical dimensions of the research and response ^3^. Engagement with these issues is essential to the success and sustainability of epidemic response and preparedness ^4^.

During the Ebola and Zika pandemics, there were concerns that the social sciences, including empirical ethics, were neglected relative to epidemiology and basic science ^5– 8^. Limited funding meant that much of the research was too reactive and rapidly conducted to contribute meaningfully to the response, in turn perpetuating low prioritization of social science research. The empirical ethics research that was conducted highlighted the practical and ethical challenges of producing quality data during a pandemic, including logistical difficulties and concerns of power dynamics between local and international actors, and between researchers and research participants and communities. These past epidemics also shone a light on the need to strengthen governance and accountability of the public health response and research during epidemics, including ensuring meaningful involvement of relevant stakeholders, including the communities affected ^1, 4, 8, 9^.

Given the scale and nature of the COVID-19 pandemic, the focus of global and national response has been on clinical medicine and public health activities. From the outset in part learning from past epidemics research and implementation networks advocated for local contexts to be considered in public health responses ^10, 11^. It was emphasized that failure to see outbreaks as political and socioeconomic emergencies, as well as public health emergencies, risked the pandemic response doing more harm than good (see for example materials on the Behavioural, Environmental, Social and Systems Interventions ( BESSI) and Training And Resource Centre ( TARSC) sites). Social justice concerns specifically were highlighted, with COVID-19 being seen to be deepening socio-economic inequities locally, nationally, and globally ^12, 13^ The urgency of conducting research was recognized as a moral issue, in line with the WHO’s position that “ *During an infectious disease outbreak there is a moral obligation to learn as much as possible as quickly as possible, in order to inform the ongoing public health response, and to allow for proper scientific evaluation of new interventions being tested*” ^14^. Significant COVID-19 specific research funding was announced, including specifically for social science research, which could also allow for empirical ethics and engagement with communities (see for example SSRC, Wellcome Trust and African Academy of Sciences). Nevertheless, the pandemic brought new challenges for research and engagement, including far more widespread travel and interaction restrictions than experienced in previous epidemics. Also, some have argued that ethicists and policymakers focused too little on the ethics of research; that is, the normative work required around risk/benefit analyses, issues concerning informed consent, and privacy considerations as well as empirical analyses of data on patient and public priorities, and the real-world barriers faced by researchers and oversight committees ^14^.

In this paper we share our experiences during COVID-19 as a pre-existing cross-site empirical ethics network, called the Global Health Bioethics Network (GHBN). We describe the ways in which GHBN members were already embedded across diverse research institutions in Africa, Europe, and Southeast Asia, and the opportunities and challenges we faced in contributing to COVID-19 responses and conducing COVID-19 empirical ethics research. Throughout, we provide illustrations of activities and experiences from across the sites, but do not seek to make comparisons across hugely diverse contexts and initiatives.

## Network description and methodological approach

### What is the Global Health Bioethics Network

The Global Health Bioethics Network (GHBN) is a collaborative partnership bringing together interdisciplinary ethics, engagement and social science teams based at the five Wellcome Africa and Asia Research Programmes (AAPs) in Kenya, Malawi, South Africa, Thailand, Vietnam, and the Ethox Centre in the UK. GHBN was established in 2012 through a strategic award provided by the Wellcome Trust. Network members meet annually for a week long ‘summer school’ and engage biannually through regional meetings. The network also encourages cross cutting learning through funding small grants, exchanges, and supporting submissions of larger collaborative projects for separate funding, such as the REACH ^15^ and CONSENT ^16^ collaborations. The AAPs themselves were established in the 1970’s, 80’s and 90’s with a focus on medical research into tropical diseases relevant to their regions (such as HIV, TB, malaria, and others). All have evolved into large inter-disciplinary research programmes over the decades, with numerous strong regional and global collaborations. All have strong links with, or are embedded within, their respective national health and research systems. For example, the Oxford University Clinical Research Unit (OUCRU) in Vietnam was co-founded in 1991 by the Hospital for Tropical Diseases (which is under the direction of the Ministry of Health) and is in the hospital grounds in a building jointly funded by the Vietnamese government and Wellcome Trust. The Malawi-Liverpool-Wellcome Trust Clinical Research Programme (MLW), set up in 1995, is an integral part of the University of Malawi College of Medicine, and has a specific Policy Unit that partners with key stakeholders in the health sector in Malawi and internationally. All the AAPs have staff involved in advisory committees locally, nationally, regionally, and globally. All also have a long history of developing and implementing community engagement activities linked to their research programmes.

The GHBN has its origins in the AAPs’ increasing commitment to addressing the ethical aspects of their research programmes and of ethical questions in relation to global health more broadly. Embedded in the Wellcome Africa and Asia Programmes, and in collaboration with the Ethox Centre at the University of Oxford, the GHBN has three main aims: (i) to promote and support the identification of ethical issues and ethical reflection across the AAPs and their research partners in a wide range of other low-resource settings; (ii) to build the capacity of the AAPs and their partners to identify and address ethical issues in their research, through post-docs, PhDs, bursaries, and other training activities; and, (iii) to facilitate and support ethics research on pressing ethical issues arising in the scientific research programmes of the AAPs and on key questions in global health.

The members of the GHBN come from many different backgrounds. While some are trained in philosophy and research ethics, others are sociologists, psychologists, human geographers, anthropologists and clinicians. There are public health and community engagement specialists. Most members who are based in the AAPs have primary roles either as research staff or community engagement personnel, with many wearing several of these professional hats. All members interact in their daily lives with a wide range of research, health system and community stakeholders, and all are actively engaged in research and engagement activities related to global health ethics. As part of their academic citizenship, members are involved in a wide range of activities including teaching, mentorship, journal editing and membership of research ethics and oversight committees as well as review boards and panels for research and international development funders.

The ways in which GHBN members are embedded within their own institutions differs, in part related to the varying ways in which social science, ethics support, and community engagement activities have evolved within those institutions; an evolution influenced at least in part by GHBN. For example, at the KEMRI-Wellcome Trust Research Programme (KWTRP), coordination of engagement activities from 2005 was initially led by a small social science group, and run as an action research activity, until the increased scale and breadth of both the engagement and social science research led to each ‘area’ functioning relatively independently. Most KWTRP GHBN members are now part of a Health Systems Research Ethics Department, with researchers conducting empirical ethics, biosocial and health policy and systems research, often in collaboration with clinical, epidemiological and bioscience colleagues in other departments. A somewhat different evolution is seen in the Thailand AAP, also known as the Mahidol Oxford Tropical Medicine Research Unit (MORU). Community engagement activities had always been conducted for studies in MORU, but it was not until the GHBN was established that this work begun to be published, contributing to the formalization of a programme of bioethics and engagement research, and ultimately the establishment of a new group called “Bioethics & Engagement” in 2015. This group has embedded ethics and engagement work into many projects at MORU such as the mass antimalarial drug administration project in the Greater Mekong Subregion ^17, 18^, in many cases with ring-fenced budgets.

### Covid-19 and how it unfolded in GHBN settings

**Figure 1.**
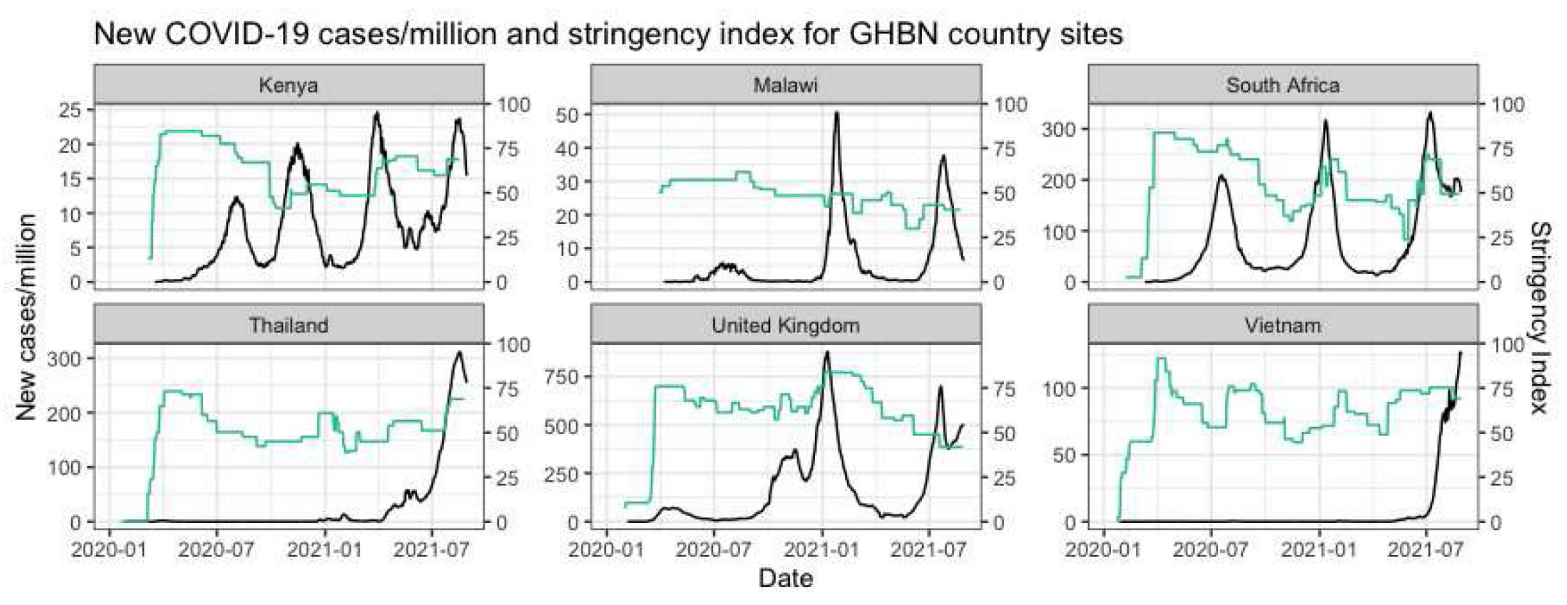
New COVID-19 cases/million and stringency index for GHBN country sites. Green line represents the stringency index and the black line represents the COVID-19 cases/million (add cite) Sources: Hale *et al.*, 2021 and Ritchie *et al.*, 2020. • *Hale, Thomas, Noam Angrist, Rafael Goldszmidt, Beatriz Kira, Anna Petherick, Toby Phillips, Samuel Webster, Emily Cameron-Blake, Laura Hallas, Saptarshi Majumdar, and Helen Tatlow. 2021. “A Global Panel Database of Pandemic Policies (Oxford COVID-19 Government Response Tracker).” Nature Human Behaviour 5(4):529–38.* • *Ritchie, Hannah, Edouard Mathieu, Lucas Rodes-Guirao, Cameron Appel, Charlie Giattino, Esteban Ortiz-Ospina, Joe Hassell, Bobbie Macdonald, Diana Beltekian, and Max Roser. 2020. “Coronavirus Pandemic (COVID-19).” OurWorldInData.Org.*

By the time COVID-19 began to be recognized across the world as a global health emergency, the GHBN had already been in place for nine years. The first COVID-19 cases were reported in different months across the main countries involved, with Thailand being the first country to record a case, followed by Vietnam, UK, South Africa, Kenya, and Malawi ( Figure 1). Notable was the very different initial responses taken by national governments in our respective countries. In UK for example, there was initial hesitation to introduce lock- down measures, and re-assurance of the public that COVID-19 was relatively mild and ‘flu like’, until mortality rates soared in April/May 2020 ^19^. In contrast, in South Africa and Kenya, as in many African countries, there were swift responses in March 2020 into hard lockdowns with the first cases identified in the two countries; responses that were initially praised in the local and regional media as highly effective. Inevitably, as the pandemic progressed over time and space, there have been shifts in the epidemiology and responses, with each country having experienced one to four distinct waves.

As with so many people around the world, GHBN members had to start to work from home with little notice when lockdowns were introduced in their countries. By April 2020, almost all countries were in varying degrees of lockdown, and most network members were working from home. Almost all non-COVID-19 studies across all sites were required by national governments, ethics review committees, or by institutional leads, to close, ‘or reduce to minimal carefully socially distanced activities, with exceptions being studies conducted online. As lockdowns hardened, only studies where participants or family members would be put at risk by a discontinuation or break in the research activities were permitted with even minimal interaction (such as clinical trials where safety monitoring was still essential). The latter monitoring moved online wherever possible.

### Tracking opportunities and challenges

Early in the pandemic we recognized the huge ethical implications of COVID-19 for health systems, communities and health research in our different contexts, and the potential value of having a network with diverse members already embedded in different settings. However, we also began to face a wide range of concerns and frustrations linked to our embedded positions. We recognized the pressing importance of exploring the ethical issues associated with COVID-19 related research, medical interventions, social distancing, and other non-pharmaceutical measures such as contact tracing. As recognition of shared issues, worries and the need to learn from one another became clear across the network, as well as our interest to contribute to responses locally, nationally, and globally, we began to organize online monthly meetings. These meetings typically lasted one to two hours, were relatively informal and relaxed, and usually involved each site outlining ongoing activities and issues to prompt discussion and learn from each other’s experience.

In the following sections we describe the opportunities and challenges we discussed over the year and a half since establishing the meetings, related to four inter-related areas: the immediate and direct responsibilities to support frontline colleagues; advisory roles in COVID-19 responses; engaging meaningfully with communities; and con- ducting empirical ethics and ethical analysis to contribute to the pandemic response. These opportunities and challenges were identified through reviewing the minutes and notes from our regular meetings over a period of one year (April 2019 to July 2020), and two cross-network discussions each of two hours (July 2020 and July 2021) where we shared the content of this paper and sought further reflection.

Throughout the following sections we note the value of being a network. In the final discussion we reflect on the benefits of the network for promoting cross disciplinary and cross context deliberation while also managing associated challenges and tensions.

## Opportunities and challenges faced by GHBN members

### Immediate and direct responsibilities with regards to supporting frontline colleagues

Emerging early in the pandemic was a concern about the health and well-being of our research team members (including field staff, community liaison staff, students, and early career researchers), the health workers and community representatives we work alongside, and family and friends.

For staff employed by our own institutions, we were fortunate that most institutions were able to continue to pay salaries while staff worked at home. However, we all recognized the different home environments that we were working in. Although there were efforts to ensure that all staff had access to computers and modems that could be used at home, and pre-paid data packages, we were never able to resolve the issues that many – but by no means all – staff were facing. For example, many GHBN members or people they were responsible for were facing challenges with electricity and internet access, inadequate or overcrowded accommodation, and multiple disruptions from family members. Other colleagues were relatively isolated, with their well-being difficult to judge through phone and online engagement. Regular communication systems and structures were set up, and new activities organized online such as journal clubs, webinars and regular informal teas or coffees to maintain a sense of community. Such initiatives have shifted over time, as the pandemic has extended. However, as noted globally, these approaches have worked better for some staff than others, and it remains difficult to ensure that those who need support are receiving it.

GHBN members wished to support colleagues in facilities and communities, but often felt powerless to do so in a systematic way. Many of us also felt a weight of responsibility given our relatively privileged position in terms of, employment security and access to healthcare and vaccination. There were some opportunities to draw upon our past activities to share the issues we were learning about across our institutions and more widely. Thus, for example in Kenya we drew on long-term health systems work ^20– 23^ and on-going interactions ^24^ to develop a set of policy briefs, on the COVID-19 implications for health system resilience and the need for better practical and emotional support for front- line health workers. The latter was discussed in a wider webinar, also involving GHBN colleagues in Vietnam, who offered insights from a very different context. Another example is from Malawi where we contributed to a policy brief on strategies for enhancing community engagement in Covid-19 case-finding, contract tracing and case referral. These policy briefs were developed with local and in-country partners and colleagues at each site. Members discussed the content of the briefs during the bi-weekly GHBN meetings, making it possible to draw upon global perspectives and contextualize policy briefs. Nevertheless, these initiatives inevitably felt piecemeal and inadequate.

### Playing advisory and reviewer roles in COVID-19 responses locally and globally

Many GHBN members felt an immediate responsibility to contribute to public debate, and to local, national, regional and global responses. Community engagement and research related opportunities and challenges are described next. Here we discuss the broader advisory and review roles many members began to play.

As noted above, GHBN members come from diverse disciplinary backgrounds, and play multiple roles within their institutions and communities, nationally and globally. Many were therefore either already in a position, or were soon offered opportunities, to take on formal advisory/review roles. These roles included: sitting on advisory groups for the South African and UK governments (through a national communications cluster and the Scientific Advisory Group for Emergencies (SAGE), respectively), and, at regional level, on the WHO-Afro African Advisory Committee on Health Research and Development (AACHRD). At the global level, GHBN members sit on the WHO Task force on social science research, the WHO Task Force on Good Participatory Practices in Emerging Pathogens (GPP-EP), the WHO Ethics Advisory Group on COVID-19, PREPHEN (a WHO led preparedness, research and response network to support epidemic ethics) and various Coalition working groups such as the Data Sharing ^25^ and Ethics Working Groups. Many members took on a substantial task in reviewing the large number of covid-19 applications submitted for funding or scientific/ethical approval. 

WHO task forces and networks have contributed to identifying COVID-19 research priorities globally, and to documents to guide Good Participatory Practice in Covid-19 clinical trials ^26^. Task force members’ drew on GHBN ideas and documents, including the policy briefs mentioned in the previous section.

Throughout the COVID-19 pandemic, these advisory roles have been important opportunities to share learning, ideas and outputs from our own sites, and from the cross-network discussions and ideas. Members recommended each other to sit on different advisory groups and networks, based on a recognition of the value of colleagues being embedded within the AAPs, having worked with government bodies, and that individuals would be able to contribute based on learning and experience from across the network.

There were dilemmas associated with our efforts to have a positive impact on the unfolding context, including: 1) how much power we really had as individuals within these groups and committees, and the power of those for a, to make a meaningful and lasting impact; 2) our recognition of the unprecedented, complex and constantly shifting nature of the pandemic and so concerns about the relevance of our past data and knowledge; and 3) the opportunities and challenges associated with community and public engagement, and conducting empirical research, described next.

### Responsibilities and approaches to engaging with community members

A central component of much of the empirical ethics work across our institutions, which over time has been valuably supported, critically reviewed, and deepened through the GHBN, is engagement with community members and broader publics ^27– 37^. An immediate potential opportunity and need under COVID-19 was to work with established community engagement networks with whom we had long term relationships to:

1) learn about community members’ and the broader publics’ priorities and concerns in relation to the response, and to communicate these through institutions and to national and global policy makers to inform locally tailored information giving and action;

2) seek community members’ advice and inputs on how to manage closure or appropriate continuation of non-COVID-19 related studies; and

3) engage community members on prioritizing and planning COVID-19 studies.

Across our different institutions we were able to work towards these aims in different ways. In terms of feeding community member and broader public priorities and concerns into information giving and action, at the most basic level GHBN members’ institutions in all countries were able to draw upon their networks and use their resources to support the national health teams to distribute government developed IEC materials. We were also able to share learning across the network in terms of what works and what does not with regards to approaches and strategies for material development and negotiating meaningful community engagement. We exchanged ideas on how to operate within different contexts, how to overcome constraints and work with opportunities and drew on each others’ materials. In some sites (for example KWTRP), all information sharing and public engagement by institutions and organisations was initially stopped, to minimise public confusion and misinformation. National requests for support from researchers focused instead on COVID-19 testing and advisory roles based on epidemiological, health systems and existing socio-behavioural data and literature. In most of the other sites, research groups were able to be more directly involved in local information sharing about COVID-19. In MLW and AHRI for example, engagement staff assisted the District Health Office and National Defence Force in organising mass awareness campaigns, including through implementing education sessions at markets, and working with traditional health practitioners and tribal/local leaders who have an influence in communities. They were also able to work with ministry of health colleagues to use social media, theatre shows, mobile vans, radio and TV stations to reach large numbers of people to provide information and address rumours and concerns. In OUCRU, GHBN members conducted media monitoring in Vietnam, Indonesia and Nepal. They started monitoring local social media and news platforms early in the pandemic (April 2020), and by continuing to do so through vaccine initiation and roll-out (starting January 2021), were able to track and respond to emerging COVID-19 anxieties with locally tailored, evidence-based social media posts. At MORU, GHBN members based on the Thai-Myanmar border engaged with Karen and Burmese migrant workers who faced language barriers regarding COVID-19 related information conveyed in Thai by the Thai authorities.

With regards to seeking community members’ advice and inputs on how to manage the sudden closure or continuation of non-COVID-19 related studies, community engagement activities often either had to stop, or to shift online, in some cases with very little notice. In Kenya for example there was little opportunity to develop and test new ways of engaging before face-to-face meetings with community representatives were halted. Over time, online approaches were developed across sites such as setting up WhatsApp groups and phone- based interactions and discussions. Although this allowed some continuation of community engagement activities, including to develop and plan COVID-19 studies, many community members have poor access to appropriate phones, if they have any phones at all, and are living in areas with intermittent electricity and poor network coverage. Even when data pack- ages are provided for community representatives, and there is electricity, technological challenges remain in terms of connectivity, influencing which community representatives can connect online with community engagement staff. Thus, there were concerns that existing challenges in hearing the voices of the most vulnerable and least connected community members would be exacerbated.

Where online interactions and face-to-face meetings were possible with community representatives, engagement staff across sites faced dilemmas in how to respond to the numerous issues raised about access to testing and health care, and about socio-economic and health consequences of COVID-19. Although community engagement staff were able to respond with government approved information, many reported feeling relatively helpless, and often faced similar concerns themselves (described further below).

As network members we drew on all of this experience, on our conversations with community members, and on our cross-site reflections to write commentaries and blog pieces to raise awareness of the issues faced, and to feed into webinars and advisory groups (including WHO guidance on GPP in COVID-19 research, as described above). Through these mechanisms, GHBN members highlighted the importance of community engagement, as well as some of the opportunities, challenges, and social justice concerns with national and global responses (see for example ^2, 38– 40^).

### Need and ability to conduct empirical ethics studies

Many of us were keen to build on our existing relationships, networks and platforms – and in particular on the GHBN - to initiate COVID-19 studies, or to add a COVID-19 lens into existing studies. We saw the need for both stand- alone studies (for example social justice implications of the response, how to give voice to the needs of the most vulnerable groups, or moral/ethical dilemmas being experienced by research and frontline staff and highlighting support needs), and for building empirical ethics elements into clinical and epidemiological studies that were rapidly being designed and implemented by colleagues (for example to incorporate explorations of community perceptions of COVID-19 and of possible COVID-19 interventions and responses). We recognised the potential of cross-site empirical ethics research, given the similarities and differences across sites in terms of the COVID-19 epidemic and responses, institutional histories/functioning, and socio-economic and political context. It was clear through our regular meetings across the network that all of our environments are shaped – albeit in different ways - by intersecting structural influences of poverty, patriarchy, capitalism, racism, and colonialism, and that most of our institutions have at least a historical dominance of biomedical approaches.

We had some unique opportunities with regards to developing, funding and conducting empirical ethics research. First, our community engagement, described above, was an opportunity to systematically document, track and share community priorities and concerns, and the ethical dilemmas and concerns of frontline research and health system staffs. Second, there was already a wide range of ongoing approved studies in place before COVID-19, where a COVID-19 lens could be added. Examples included epidemiological and clinical studies, and studies evaluating community engagement, exploring community treatment-seeking behaviour, identifying frontline health worker priorities and concerns, and examining system governance and oversigh. Many of these studies involved GHBN members. For studies led by members, we discussed in our network meetings that adding a COVID-19 and community perspective lens to these studies was potentially feasible where interviews could be conducted online or following local social distancing rules. Online interviews, particularly for interviews seeking more than simple quantitative data, were most realistic to consider for longitudinal studies where relationships were already in place between researchers and participants. Third, we were able to design and conduct new studies, considering social distancing rules and the potential for these to change over time. All amendments to studies and new studies required institutional and national ethics approval, following careful consideration of potential ethical issues (including risks/benefits, consent, confidentiality, and data safety/protection). We had regular discussions across the network on how to do this under COVID-19 restrictions and in the face of emerging new national and international guidance.

In terms of funding, we were fortunate to have costed salary extensions offered automatically by one of the main funders in our institutions – the Wellcome Trust. This allowed for an extension of the GHBN itself, and for some studies to be initiated or continued with relatively little additional funding. A valuable source of additional funds was the small, competitively run bursary scheme run by GHBN for early career research in LMICs; a scheme that has been in place from the beginning of the network. An example of an international study funded through a combination of mechanisms is provided in Box 1. As elsewhere, the social scientists involved in planning studies sometimes had to make methodological compromises to fit national or institutional rules (such as more on-line and less in-depth deliberative work, leading to concerns about missing the voices of the most vulnerable), and - where face-to-face interviews were permitted - worried about team members’ safety. Our dilemmas and discussions fed back into decision-making across our institutions and more widely, as described above.

**Box 1. Examples of funded international studies**

One example of a study that received both internal and external funding is the Social Science and Public Engagement Action Research in Vietnam, Indonesia and Nepal (SPEAR) study, initiated in June 2020. The primary aim of SPEAR was to explore the experiences and impact of COVID-19 and the public health response for healthcare workers and related staff from a variety of healthcare settings, as well as community members who were more impacted by COVID-19 and/or the response. The study was conducted in 12 sites across the three countries, each of which were facing different reported levels of COVID-19 and different responses. Each site had both healthcare worker and community participation and each country had representation from both rural and urban communities. For data collection, the team used media monitoring, questionnaires (self-administered online or paper and interview administered in person or on phone), in-depth interviews (in person and online), and digital diaries. In January 2021, the SPEAR study was expanded to include an additional focus on access to and perceptions of COVID-19 vaccination within all community sites involved in SPEAR. Another example of an externally funded proposal is a multi-site study – involving researchers based in Kenya, South Africa and Ghana - aimed at examining how research review and regulatory systems have responded to the high demand for COVID19 research, including how systems have been changed, the (ethical) issues raised, the opportunities and challenges from the perspective of different stakeholders, and if and how any challenges have been addressed.

A challenge faced by many GHBN members and colleagues for COVID-19 related work in many (but not all) sites was lengthy funding and especially ethics review processes. For example, although the SPEAR study ( Box 1) was approved within three months of submission, a different study in another country (an interview and observation-based study aimed at systematically exploring the ethics issues and dilemmas experienced under COVID-19), took 8 months from submission to final approval, primarily due to administrative delays and changes in regulatory processes. Further challenges for empirical ethics included, particularly early in the pandemic, keeping pace with biomedical research, for which there was often a clearer and more immediate demand from national stakeholders. Broader challenges included a relatively small group of people being aware of the unfolding requirements within institutions, and a fast-evolving situation which was not easily shared across relevant colleagues. This was due to the siloed nature of the different disciplines in several network institutions where ethics researchers were not involved and included in the fast-paced biomedical research response. The changing nature of the pandemic also made it challenging for current information to be shared as the situation could change overnight.

Findings from social science/empirical ethics studies that were prioritized and conducted have begun to be published and shared through our networks, contributing to public and policy maker awareness about lived experiences, and about priorities and concerns on the ground ^13, 31, 41– 45^.

Finally, in addition to the empirically driven ethics research that the GHBN was able to undertake during the COVID-19 pandemic, some members of the network were also well-placed – because of their membership of GHBN, their embeddedness in COVID-19 related research initiatives, and their policy roles – to write a number of influential papers and blogs addressing key ethical questions relating to the impact of the pandemic and responses to it ^43, 45– 47^.

## Discussion

The Global Health Bioethics Network is a strong and long-term social science and ethics network bringing together researchers and practitioners from Africa, Europe, and South East Asia. The GHBN is embedded in multi-disciplinary research programmes and in national health systems, and has long-term funding from the Wellcome Trust covering both the network itself and the core sites involved. This has allowed us to interact in ways that are different to some of the concerns typically raised about ‘parachute’ style international collaborations ^5, 48^, where Northern researchers drop into Southern settings to undertake research without adequate attention to equitable treatment of Southern partners and understanding of local contexts. In response to the pandemic, GHBN offered us important opportunities for contributing to COVID-19 research, but as with many other researchers across the world, we also encountered many tensions and challenges.

As an existing network with established relationships, many of us discussed feeling able to share issues and concerns in a safe and supportive online environment. Given the difficult and uncertain circumstances of the COVID-19 pandemic, these informal debrief sessions were referred to in discussions as a valuable form of ‘group therapy’ , (similar to but less formalized than fora described by Molyneux *et al.* ^49^. and McMahon and Winch ^50^. All of us described feeling reassured through these discussions and the sense of community they created. For example, the regular sessions helped us feel that our issues and concerns were shared by others and legitimate, even where our own institutions were unable to hear or engage with them.

The network also offered us intellectual, emotional and practical support to reflect upon and discuss our various responsibilities, not least balancing across our (sometimes competing) responsibilities to safeguard the wellbeing of staff and colleagues, engage with community representatives and other stakeholders, conduct timely and responsive research and offer relevant and appropriate advice at different levels. Being embedded long-term offered us opportunities in terms of deep institutional and contextual knowledge and having existing relationships with diverse stakeholders. This gave us access to community members, health and research institution staff and policymakers. Having these established relationships in place gave us opportunities to understand and work with those with the power to make decisions in complex environments. Being networked offered us opportunities to draw upon a wide range of roles and expertise operating in multiple contexts, and to bring together perspectives internal and external to institutions (i.e. allowing us to benefit from insights based on different positionalities ^51^). This in turn supported us to consider similarities and differences across contexts and gave us greater confidence to contribute to institutional, national and global discussion and debates (see for example resources shared on Global Health Bioethics Network and on The Global Health Network).

Many tensions and challenges remain, as summarized in Table 1. In terms of conducting empirical research, we faced the challenge that has been shared elsewhere of social sciences, including empirical ethics, sometimes being considered as ‘nice-to-have’ rather than as essential ^5– 8^). Therefore, unlike clinical or epidemiological research, for example, empirical social science and ethical research was commonly regarded as something that can wait until the urgency of the situation has passed to be planned, reviewed and conducted. Where the value of social science was seen, we noted a preference for quantitative and representative data, and for rapidly produced research, as opposed to more in-depth and time-consuming qualitative data investigating context, values, beliefs, concerns, solutions, and lived experiences. The importance of conducting a range of research across time lines was highlighted from early on in the pandemic on social media and through research networks (see for example Marquette H, April 2020). However, this was challenging to implement in practice. We were all aware of and conflicted by - on the one hand - a desire to help with the response through research, and on the other a concern that the research that we were conducting should be relevant, appropriate and of high quality. A particular concern was being careful not to turn our full attention to COVID-19, thereby undermining our ability to conduct essential research on other important social and health issues in communities. Another concern expressed by some network members was not feeling experienced enough to contribute to debates on ethical issues around COVID-19 related decision-making; an area we wish to continue to teach one another across the network.

**Table 1.** Tensions and issues faced.

Tension/issue	How tension was felt
*Social science vs ‘real’ science*	Social science is sometimes seen as non-essential research to do later, once the more critical epidemiological or clinical research is underway
*Quantitative vs qualitative* * social science*	Quantitative social science can be prioritized over more in-depth qualitative work, and studies with small sample sizes dismissed or not taken seriously (even when appropriate for the question, theoretically informed, and well implemented)
*Speed vs quality*	Producing findings ‘NOW’ vs learning in depth and in detail for future
*In-depth, inclusive learning*	‘What is ethical?’ and what is possible under physical distancing rules
*Community engagement vs* * social science*	Engagement and social science are always the same activities, even though there may be overlaps in people, methods, and interests
*Confidentiality vs impact*	Protecting participant/institution confidentiality vs maximizing the policy impact/uptake of research findings
*Immediate vs longer-term/* *structural issues*	Balancing dealing with immediate challenges/issues against tackling longer-term/structural issues such as having to seek funding primarily from high income countries
*Social science findings vs* * stakeholder interests*	Some findings can be uncomfortable for some research stakeholders ( *eg* research institution/ biomedical research team / government)… requires management of relationships

A related tension evolved around the similarities, differences and overlaps between community engagement and social science research, including empirical ethics. Engagement activities offer opportunities to answer research questions, but research may have particular methodological and ethical considerations (for example regarding consent, benefits and confidentiality) and usually requires different institutional and national approval processes. We had to consider these similarities, differences and their implications in all of our activities. For example, with the SPEAR study described above, the integration of engagement and social science provided an opportunity to use findings from engagement activities such as participant-led films or ‘digital diaries’ and media monitoring to inform the survey and interview tools at the early stages of the project ^13, 31^.

During the social science data collection, especially related to vaccines, the engagement and social science teams met regularly so that data from the interviews could inform engagement messages.

Across both engagement and research activities involving community members, there was a tension in ensuring that the voices of those most vulnerable to the negative impacts of COVID-19 were heard, while also ensuring that the activities themselves did not perpetuate those vulnerabilities. Thus, for example in KWTRP, the team/members considered building on existing relationships with mothers of young children in rural and urban settings discharged from hospital ^52, 53^ to learn about their priorities and concerns regarding COVID-19. However, during GHBN and team meetings we agreed there was too much potential to inadvertently contribute to disadvantages and harms, both physical (COVID-19) and in terms of perceived responsibilities to act upon issues raised that could not be resolved.

A final tension and challenge concerns the recognition that the institutions most centrally involved in the GHBN are all relatively privileged compared to many other institutions based in low-resource settings, and that GHBN members within those institutions were unequally involved in the regular COVID-19 related discussions (given differences across staff in terms of home living arrangements, access to electricity/power cuts, health and well-being, and levels of work and home-related responsibility). Although the network activities and ways of working seek to challenge such inequities, our own interactions are also shaped – albeit in different ways – by intersecting structural influences of poverty, patriarchy, capitalism, racism, and colonialism that interplay to impact on us all differentially over time and space. As a network this is an area we will continue to reflect upon and work towards positive transformation.

## Conclusion

A lesson from this difficult and unprecedented time is that embedded ethics has an important role to play in effective infectious diseases research and response. Notwithstanding the limitations, practical concerns, and difficulties outlined above, it is our strong view that funding of an embedded ethics network that brings together ethicists and social scientists from a wide range of contexts in a sustainable, long-term, networked and globally distributed collaboration has the potential to be a crucial resource in pandemic preparedness, resilience, and response. In light of the ongoing COVID-19 pandemic, and the likelihood of future pandemics and other global health emergencies, the embedded work of networks such as the GHBN continue have great potential.

## Data Availability

No data are associated with this article.
