# Supplementary material for: A network of empirical ethics teams embedded in research programmes across multiple sites: opportunities and challenges in contributing to COVID-19 research and responses
Source: Wellcome Open Res. 2023 Jan 27;7:48. Originally published 2022 Feb 10. [Version 2] doi: 10.12688/wellcomeopenres.17548.2 (PMC10457565; doi:10.12688/wellcomeopenres.17548.2)
Supplement: Peer review history

# Peer review: Reviewer response for version 2

**DOI:** 10.21956/wellcomeopenres.20829.r65209

**Published:** 2023-08-25

**Authors:** Lario Viljoen

**Affiliations:** 1 Desmond Tutu TB Centre, Department of Paediatrics and Child Health, Faculty of Medicine and Health Sciences, Stellenbosch University, Cape Town, South Africa

Thank you to the authors for this interesting contribution in an often-overlooked component of health research. The reflections on pandemic research from a bio-ethics group brings a fresh perspective to the plethora of COVID-19 literature available. The piece carries even more value as the contributions are from diverse contexts and the practical examples make the article easy to digest. Some minor comments below:

 It might be worth mentioning in the introduction the risks faced by research fieldworkers (not only medical researchers) in doing data collection during the pandemic – and the ethical implications thereof (end of paragraph 3).

 The background section on the GHBN is well written and helps the reader to understand the context.

 On the "tracking opportunities" section, the authors should indicate what is the justification for reviewing minutes of meetings for this specific manuscript 6 months prior to when the first case of COVID-19 was reported.   

 In the discussion section, the authors do well to draw the article together. However, there is some conflation between social science research in general and investigations/explorations of ethics - and although the two are definitely connected, it should be clear that understandings of ethics/ethical concerns is the focus of the article. The authors draw out the distinction between community engagement and social science and a sentence doing the same for social science research and 'ethics' could be beneficial. I understand that members of the GHBN can wear both hats - which makes it complicated to explain. 

 In the conclusion section, how do the authors propose that ethical explorations be included/considered alongside biomedical responses during potential future global epidemics? 

Does the article adequately reference differing views and opinions?

Yes

Are all factual statements correct, and are statements and arguments made adequately supported by citations?

Yes

Is the Open Letter written in accessible language?

Yes

Where applicable, are recommendations and next steps explained clearly for others to follow?

Yes

Is the rationale for the Open Letter provided in sufficient detail?

Yes

Reviewer Expertise:

Public health, socio-behavioural research, HIV, paediatric TB, community health

I confirm that I have read this submission and believe that I have an appropriate level of expertise to confirm that it is of an acceptable scientific standard.

# Peer review: Reviewer response for version 2

**DOI:** 10.21956/wellcomeopenres.20829.r54545

**Published:** 2023-02-03

**Authors:** Abha Saxena

**Affiliations:** 1 Independent Bioethics Advisor, Geneva, Switzerland

I have just reviewed the revised paper, and would like to thank the authors for taking seriously my advice and recommendations. I am very happy with the revised version of the paper, which lays out the context and the background very nicely now, and have no hesitation in approving this version of the paper as submitted.

Does the article adequately reference differing views and opinions?

Partly

Are all factual statements correct, and are statements and arguments made adequately supported by citations?

Partly

Is the Open Letter written in accessible language?

Partly

Where applicable, are recommendations and next steps explained clearly for others to follow?

Partly

Is the rationale for the Open Letter provided in sufficient detail?

Yes

Reviewer Expertise:

Global health ethics, research ethics, public health and health systems ethics

I confirm that I have read this submission and believe that I have an appropriate level of expertise to confirm that it is of an acceptable scientific standard.

# Peer review: Reviewer response for version 1

**DOI:** 10.21956/wellcomeopenres.19404.r48656

**Published:** 2022-03-01

**Authors:** Abha Saxena

**Affiliations:** 1 Independent Bioethics Advisor, Geneva, Switzerland

The authors share the experiences, during 2 years of the COVID-19 pandemic, of a pre-existing cross-site multidisciplinary ethics network-the Global Health Bioethics Network-which brings together researchers and practitioners from Africa, Europe, and South East Asia. The bioethics network, funded by the Wellcome Trust, exists within the context of Wellcome Trust funded Research Programmes on tropical diseases and has existed as a network for over eight years. 

 The authors describe the impact of the pandemic on their work at a centre level and at the network level, how the network responded to the ground realities of the pandemic, the challenges they faced, and how and why the network was able to respond effectively and meaningfully to the pandemic related debates at the global level. But the paper does not do a very good job of demonstrating how the output of the network was more than the sum of the output of individual centres. The authors make the claim that it is a good idea to have ethics embedded in research programmes, which could be read as being a recommendation, but they do not share experiences of how these centres came to become embedded. Nor does it provide insights that could benefit other research centres aspiring to develop embedded ethics programmes. These aspects could be strengthened. References are required for at least one statement made in the paper. All these aspects are further elaborated in the comments below.

 While the narrative in the paper is very compelling and impressive, first of all, I would like to suggest some re-organization - the second and third paragraphs of the Discussion could perhaps, more usefully, be incorporated in the section discussing challenges and opportunities, which would explain better why the network was able to respond so effectively, and how the network members supported each other. As currently organised, the paper describes the contribution of the members of the GHBN to the global debates but does not explain how the members benefitted from being a network until much later till we come to the Discussion. The Discussion should focus only on the enabling and challenging factors, and not on the advantages and opportunities offered by the network. 

 Apart from this organisational issue, the paper raises a number of questions:

1. It would have been interesting to know more about the working of the network prior to COVID-19 - while the aims of the network are described, as well as the roles and responsibilities of individual members, we know little about how the ethics centres at the different sites were interacting or engaging with each other - was it through annual meetings, through regular exchange of information, through monthly or quarterly get togethers? was there an exchange of scholars? What were the outputs of the *network *over the 9 year period that it has existed, and how has the network benefitted the individual centres? Were the activities of the network funded by the Wellcome Trust or another funder? Such background information about the network would help us understand its contributions to the COVID-19 debates.
2. It is unclear how being in the GHBN added value to the work carried out by the individuals? Did greater opportunities come to individuals because they were part of a network (i.e. they would not have been invited to the global debate if they had not been part of the network, and being in a network was seen as a positive for contributions to the global debate more holistically), or because some of the colleagues on this network are professionally better known or more influential in global circles (and from the global perspective, being part of a network was incidental)?  
3. Related to the above, the authors describe the development of policy briefs. Were the policy briefs developed collaboratively with other network partners? if not, how did being in the network support or facilitate the development of the briefs? 
4. In the section just before the start of the Discussion, the authors mention that some members of the network were able to write a number of influential papers and blogs. Would be good to know how the authors define "influential" and would be good to cite the influential papers/blogs here. 
5. The paper describes - "under responsibilities and approaches to engaging with community members" the various ways that individual centres were/are engaging with the communities in which they are working, but it would be interesting to know if being in a network enhanced or informed their community engagement activities, and if yes, then how. 
6. The section on "need and ability to conduct empirical ethics studies" is more about embedded or empirical ethics, than a description of the impact or contributions of the network to the conduct of empirical ethics studies. This aspect should be elaborated. 
7. Would be nice to know if certain centres were able to contribute more to the global discussions than others, and if so what were the facilitatory factors. Related to that, it seems that only UK and S. Africa are mentioned as having contributed to the national discourse. If this is true, then what about the other centres? What factors operating in S. Africa and UK enabled those centres to have a broader impact than others and what are the lessons learnt? 
8. GHBN colleagues in Malawi and AHRI (the acronym should be spelt out in the paper) seem to be more effective in supporting both the district level administration and the Ministry of health in implementing mass media campaigns, education sessions, and working with tribal/local leaders, than those in other centres. A discussion on what were the facilitating factors would be helpful. 
9. Amongst one of the challenges mentioned is the "Broader challenges included a relatively small group of people being aware of the unfolding requirements and issues within institutions, and a fast-evolving situation which was not easily shared across relevant colleagues". This should be expanded - why was information sharing in a timely manner challenging and what are the lessons learnt from this experience? 
10. One of the listed messages from the paper is "Such networks have the potential to contribute to negotiating persisting tensions and challenges, including regarding power and politics in institutional responses, and the position of empirical ethics in multi-disciplinary research programmes; and can make important contributions to pandemic preparedness." The authors describe that being embedded and long term helped them to acquire deep institutional and contextual knowledge and an understanding of "how things work". However, these centres and the GHBN network itself are able to function as they do because of dedicated funding. Which brings the question of what role do funders have to fund and nurture such embedded ethics groups within the research programmes that they fund, and how other funders can learn from this experience. Some discussion on the role played by the funding from Wellcome Trust would be relevant. 
11. The article has many complex sentences - I feel that it could be written in easier to read, simplified text for better understanding, especially by those who are not native English speakers. 

Does the article adequately reference differing views and opinions?

Partly

Are all factual statements correct, and are statements and arguments made adequately supported by citations?

Partly

Is the Open Letter written in accessible language?

Partly

Where applicable, are recommendations and next steps explained clearly for others to follow?

Partly

Is the rationale for the Open Letter provided in sufficient detail?

Yes

Reviewer Expertise:

Global health ethics, research ethics, public health and health systems ethics

I confirm that I have read this submission and believe that I have an appropriate level of expertise to confirm that it is of an acceptable scientific standard, however I have significant reservations, as outlined above.
